# Escalating pyrethroid resistance in two major malaria vectors *Anopheles funestus* and *Anopheles gambiae* (s.l.) in Atatam, Southern Ghana

**DOI:** 10.1186/s12879-022-07795-4

**Published:** 2022-10-25

**Authors:** Leon M. J. Mugenzi, Gabriel Akosah-Brempong, Magellan Tchouakui, Benjamin D. Menze, Theofelix A. Tekoh, Micareme Tchoupo, Francis N. Nkemngo, Murielle J. Wondji, Ekene K. Nwaefuna, Michael Osae, Charles S. Wondji

**Affiliations:** 1 Centre for Research in Infectious Diseases (CRID), P.O. Box 13501, Yaoundé, Cameroon; 2 African Regional Postgraduate Program in Insect Science, University of Ghana, Legon, Accra, Ghana; 3 Biotechnology and Nuclear Agriculture Research Institute, Ghana Atomic Energy Commission, Accra, Ghana; 4 Vector Biology Department, Liverpool School of Tropical Medicine, Pembroke Place, Liverpool, L3 5QA UK

**Keywords:** Insecticide resistance, Atatam, *Anopheles*, Malaria, Long-lasting insecticidal nets

## Abstract

**Background:**

Aggravation of insecticide resistance in malaria vectors is threatening the efforts to control malaria by reducing the efficacy of insecticide-based interventions hence needs to be closely monitored. This study investigated the intensity of insecticide resistance of two major malaria vectors *An. funestus* sensu stricto (s.s.) and *An. gambiae* sensu lato (s.l.) collected in southern Ghana and assessed the bio-efficacy of several long-lasting insecticidal nets (LLINs) against these mosquito populations.

**Methods:**

The insecticide susceptibility profiles of *Anopheles funestus* s.s. and *Anopheles gambiae* s.l. populations from Obuasi region (Atatam), southern Ghana were characterized and the bio-efficacy of some LLINs was assessed to determine the impact of insecticide resistance on the effectiveness of these tools. Furthermore, molecular markers associated with insecticide resistance in both species were characterized in the F_0_ and F_1_ populations using PCR and qPCR methods.

**Results:**

*Anopheles funestus* s.s. was the predominant species and was resistant to pyrethroids, organochlorine and carbamate insecticides, but fully susceptible to organophosphates. *An. gambiae* s.l. was resistant to all four insecticide classes. High intensity of resistance to 5 × and 10 × the discriminating concentration (DC) of pyrethroids was observed in both species inducing a considerable loss of efficacy of long-lasting insecticidal nets (LLINs). Temporal expression analysis revealed a massive 12-fold increase in expression of the *CYP6P4a* cytochrome P450 gene in *An. funestus* s.s., initially from a fold change of 41 (2014) to 500 (2021). For both species, the expression of candidate genes did not vary according to discriminating doses. *An. gambiae* s.l. exhibited high frequencies of target-site resistance including Vgsc-1014F (90%) and *Ace-1* (50%) while these mutations were absent in *An. funestus* s.s.

**Conclusions:**

The multiple and high intensity of resistance observed in both malaria vectors highlights the need to implement resistance management strategies and the introduction of new insecticide chemistries.

**Supplementary Information:**

The online version contains supplementary material available at 10.1186/s12879-022-07795-4.

## Background

Malaria remains the deadliest vector-borne disease accounting for more than 627,000 deaths annually with half of the world’s population being at risk [1]. There have been intensified malaria control and elimination efforts over the past two decades which have helped to avert about 1.5 billion cases and 7.6 million deaths [1]. However, for the past 5 years, there have been no significant reduction in the number of malaria-related cases worldwide [2]. With this stagnation in progress, the targets outlined by World Health Organization (WHO) in the Global Technical Strategy for Malaria 2016–2030 which aimed at reducing the global malaria burden by 90% by 2030 [3] may not be met.

Vector control interventions based on insecticides have been the cornerstone for malaria control and eliminations efforts as they are the most widely used with more than 2 billion long-lasting insecticide-treated net (LLINs) already distributed across the world (https://allianceformalariaprevention.com/working-groups/net-mapping/) and over 97 million people protected by indoor residual spray [2]. The efficacy of these control tools is threatened by the development of insecticide resistance in the malaria vectors against the few public health insecticides currently approved by WHO [4]. Insecticide resistance to all four main insecticide classes has been reported across the globe with pyrethroid resistance being widespread [5].

Mosquitoes can become resistant to insecticide through changes in the insecticide target preventing its efficient binding (target-site resistance); increased breakdown and elimination of the insecticide by detoxification enzymes (metabolic resistance); cuticle thickening to reduce insecticide penetration (cuticular resistance). Mosquitoes can also change their behaviour to avoid contact with insecticide-treated surfaces, a mechanism termed behavioural resistance [6]. Various *Anopheles* species have been identified in Africa and shown to differ in their ability to transmit malaria [7], as well as their ability to survive insecticide exposure [8].

To break the resistance cycle and maintain the efficacy of the current and future insecticides, insecticide resistance management strategies need to be integrated into the National Malaria Control Programmes strategic plans. As such, up-to-date data from resistance surveillance in local malaria vectors are essential in decision-making as insecticide resistance is dynamic.

Malaria remains a major public health issue in Ghana, with *Anopheles gambiae* and *Anopheles funestus* being the predominant vectors [8]. While much information is available on the insecticide resistance profile of *An. gambiae* s.l*.*, very little exists for *An. funestus* s.s. with most data originating from a gold mining town in southern Ghana, Obuasi [9, 10].

Previous studies characterized the insecticide resistance profile of *An. funestus* s.s. and reported resistance to dichlorodiphenyltrichlororethane (DDT), permethrin and bendiocarb in 2005 and later in 2014, high resistance to pyrethroids, carbamates and DDT were observed with mortality levels below 50% [10]. Metabolic resistance has been identified as the main mechanism since no mutation in the sodium channel gene associated with the target-site resistance phenotype was found [9]. The glutathione S-transferase *GSTe2* and the cytochrome P450s *CYP6P9a*, *CYP6P9b,* and *CYP6M7* previously implicated in pyrethroid resistance were found to be up-regulated [10]. Furthermore, transcriptional analysis of resistant *An. funestus* s.s. across Africa revealed that the duplicated *CYP6P4a* and *CYP6P4b* are significantly more up-regulated in Ghana [11] but their role in pyrethroid resistance has not yet been validated. Recent studies have reported increased pyrethroid resistance levels in *An. funestus* s.s. populations of Southern and Eastern Africa with mosquitoes surviving longer insecticide exposure and higher doses of insecticides [12, 13]. These escalations of insecticide resistance are more likely to lead to control failure than standard resistance levels [14] by reducing the efficacy of vector control tools which could lead to a rise in malaria incidence and fatalities. Such insecticide resistance surveillance data are lacking for *An. funestus* s.s. population from West Africa notably in Ghana. *An. gambiae* s.l. population from Ghana has shown resistance to all four public health insecticides with high-intensity resistance reported to pyrethroids and carbamates and low resistance intensity to pirimiphos methyl [15].

To fill this gap and assist in resistance management, this study aimed to extensively characterize the insecticide resistance level in *An. funestus* s.s. and *An. gambaie* s.l. mosquitoes in the Obuasi area (Atatam) in southern Ghana. In addition, the bio-efficacy of some selected new LLINs against these local mosquito populations was determined and the molecular basis of the resistance escalation was investigated.

## Methods

### Study site and mosquito collection

**Fig. 1:**
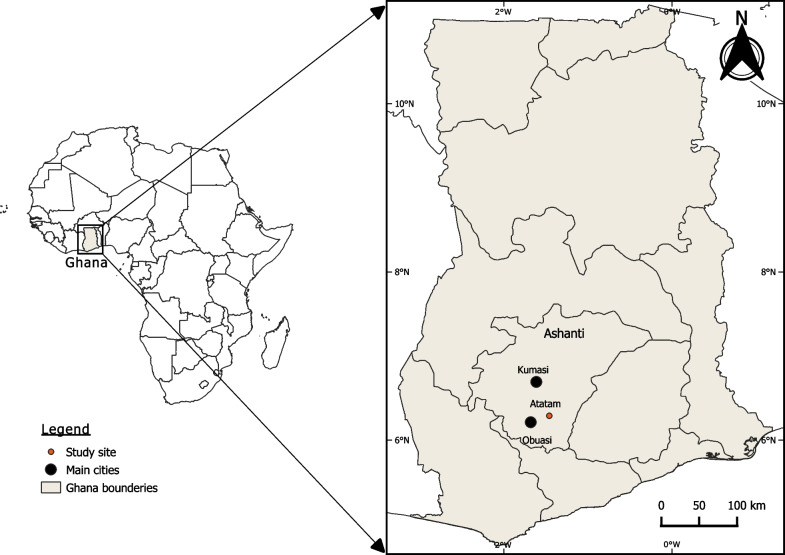
Map of Ghana showing the geographical location of the collection site Atatam

Indoor blood-fed resting *Anopheles* mosquitoes were collected in the village of Atatam (06° 17.377″ N, 001° 27.545″ W) located in the Adansi Asokwa District, close to Obuasi municipality of the Ashanti Region (Southern Ghana) Fig. 1. The area experiences a semi-Equatorial climate with an average temperature of about 27 °C and average annual rainfall ranging between 1250 and 1705 nm [16]. Most of the inhabitants are practicing cocoa and subsistent farming. Mosquitoes were collected in July (peak of the major raining season) and October (peak of the minor raining season) 2021.

Verbal consent was obtained from the village chief and house owners before starting the collections. Each collection was planned for 3–5 days, and mosquitoes were aspirated using prokopack electric aspirators. Morphological identification of the collected mosquitoes was done using the morphological keys [17]. *An. funestus* s.l. and *An. gambiae* s.l. females were morphologically identified and transferred from the cage into paper cups covered with a net and maintained for 5–7 days until they became fully gravid. The mosquitoes were then forced to lay eggs in 1.5 mL micro-centrifuge tubes and the obtained larvae were reared to adults as previously described [18].

### DNA extraction and species identification

One hundred and twenty *An funestus* s.l. F_0_ females were dissected into head plus thorax and abdomen, after which genomic DNA was extracted from each part using the LIVAK method [19]. Briefly, DNA was isolated from individual mosquitoes that were homogenized in 100 µl of lysis buffer (composed of 0.5% SDS/0.08 M NaCI/O.16 M sucrose/0.06 M EDTA/O.I2 M Tris-HCI, pH 9) and incubated at 65 °C for 30 min. Four Molar potassium acetate was used to precipitate out the proteins followed by centrifugation. Finally, the DNA was rinsed with ethanol, dried and resuspended in deionized water [19]. The cocktail polymerase chain reaction (PCR) [20] was used to identify the specific member of the funestus group collected. In addition, 65 *An. gambiae* s.l. were also dissected and molecularly identified using the SINE PCR [21].

### *Plasmodium* infection rates

The bisected mosquito head/thoraxes and abdomens were screened separately for *Plasmodium falciparum* and *Plasmodium ovale*, *Plasmodium vivax*, and *Plasmodium malariae* (OVM) using the TaqMan assays previously described [22]. Sporozoites or oocyst infections were indicated by positive head plus thorax or abdomen respectively. Positive samples were later validated by nested PCR [23].

### World Health Organization insecticide susceptibility assays

Non-blood-fed female F_1_ *An. funestus* s.s. and *An. gambiae* s.l. aged between 3 to 5 days were subjected to WHO tube bioassays [24] to determine the insecticide resistance profile. *An. funestus* s.s. and *An. gambiae* s.l. mosquitoes were exposed to discriminating concentration (DC), 5 × DC, and 10 × DC of pyrethroids [type I permethrin (0.75%, 3.75% and 7.5%) and type II deltamethrin (0.05%, 0.25% and 0.5%) and alpha-cypermethrin (0.05%, 0.25% and 0.5%)]; the organochlorine [DDT (4%)]; carbamates [bendiocarb (0.1%) and propoxur (0.1%)]; and organophosphates [pirimiphos methyl (0.25%), fenitrothion (1%) and malathion (5%)]. In each test, at least 100 females divided into four replicates of 20–25 females per tube were exposed per insecticide along with 40–50 mosquitoes (2 replicates) were used as controls and exposed to non-impregnated papers. Mosquitoes were exposed for 1 h, and final mortality was recorded after 24 h post-exposure. The impregnated papers were supplied by the WHO supplier at Universiti Sains Malaysia.

The strength of phenotypic resistance was assessed for insecticides where resistance was confirmed for the standard diagnostic doses and for which 5- and 10-times doses are available. Furthermore, a synergist bioassay with 4% Piperonyl Butoxide (PBO) was carried out to assess the possible contribution of metabolic resistance [24]. When control mortality was between ≥ 5% and ≤ 20%, Abbott’s formula was used to correct the test mortality.

### WHO cone test

The bio-efficacy of insecticide-treated bed nets against the *An. funesus* s.s. and *An gambiae* s.l. populations from Atatam were assessed using cone assays as recommended by WHO [25] with slight modification. Mosquitoes were exposed to the following standard LLINs [PermaNet 2.0, Interceptor, DuraNet, Olyset, MagNet], PBO nets [PermaNet 3.0, Olyset plus] and novel nets [Royal guard and Interceptor G2]. A piece of untreated net was used as a control. Fifty F_1_ females aged 3 to 5 days old grouped in 5 replicates were aspirated into plastic cones placed over the treated nets and exposed for 3 min before transferring them into a paper cup. Mosquitoes were provided with 10% sugar solution and knockdown and mortality were scored 1 h and 24 h post-exposure respectively.

### Genotyping of resistance markers

The L119F-*GSTe2*, *CYP6P9a,* and *CYP6P9b* mutations previously associated with metabolic resistance to DDT and pyrethroids in *An. funestus* s.s. were genotyped to identify if these markers are also driving resistance in West Africa (Ghana). The L119F-*GSTe2* resistance marker genotyping was performed using the allelic-specific PCR [26] and the *CYP6P9a* and *CYP6P9b* markers using the PCR restriction fragment polymorphisms [11, 27]. Furthermore, the GABA receptor RDL A296S and Acetylcholinesterase-1 N485I target site mutations associated with dieldrin and carbamate resistance respectively were also genotyped using the Taqman SNP genotyping assay [26, 28]. The target site mutations L1014F and L1014S *kdr* (both East and West) are strongly associated with resistance to pyrethroids and DDT [29, 30], as well as the N1575Y suggested to either compensate for deleterious fitness effects of 1014F or confer additional resistance to insecticides were also genotyped [31]. In addition, the *Ace-1* G119S resistance mutations were assayed in F_0_ *An. gambiae* s.l. mosquitoes using the Taqman technique [22]. The association of these markers with phenotypic resistance was determined by genotyping each marker in exposed F_1_ mosquitoes both in the survivor and dead groups.

### Transcriptional profile of major metabolic resistance genes

Quantitative reverse transcription polymerase chain reaction (qRT-PCR) assay was used to assess the expression profile of detoxification genes previously associated with pyrethroid resistance. This included *CYP6P9a, CYP6P9b, GSTe2, CYP9K1, CYP6M7,* and *CYP6P4a* for *An. funestus* s.s. and *CYP6M2, GSTe2, CYP9K1, CYP6P4, CYP6Z1, CYP6P3, CYP4G16,* and *CYP4G17* for *An. gambiae* s.l. In addition, the expression of the chemosensory proteins *SAP1*, *SAP2* and *SAP3* recently shown to be involved in pyrethroid resistance [32] were also assessed in *An. gambiae* s.l. The expression profile was compared between *An. funestus* s.s. F_1_ resistant to deltamethrin (1x, 5x, and 10x), unexposed and FANG insecticide susceptible laboratory strain while for *An. gambiae* s.l*.,* F_1_ resistant to permethrin 1x, unexposed and Kisumu laboratory strain were used. This involved extraction of total RNA from 3 pools of 10 F_1_ mosquitoes each using the Picopure RNA isolation kit (Arcturus) followed by cDNA synthesis and lastly qPCR [33]. The 2^−ΔΔCT^ method was used to calculate the relative expression [34] and fold changes after normalization with housekeeping genes *RSP7* (ribosomal protein S7; VectorBase ID: AFUN007153-RA) and *Actin* (VectorBase ID: AFUN006819-RA) for *An. funestus* s.s. while ribosomal protein S7 (AGAP010592) and elongation factor (AGAP005128) were used for *An. gambiae* s.l. Unpaired Student’s t-test was used to determine whether the difference observed in expression was significant.

## Results

### Species identification

A total of 603 blood-fed *Anopheles* mosquitoes were collected in July 2021 with 424 (70.32%) morphologically identified as *An. funestus* s.l. and 179 (29.68%) identified as *An. gambiae* s.l. Later in October 2021, 574 blood-fed *Anopheles* were collected with 267 (46.52%) *An. funestus* s.l. and 307 (53.48%) *An. gambiae* s.l. Molecular identification by PCR on a subset of 78 F_0_ *An. funestus* s.l. revealed that 81.4% of the samples that amplified (70/86) were *An. funestus* s.s. with a band at 505 bp in addition to 2 *An. Parensis* (252 bp) and 1 *An. Rivulorum* (411 bp)*.* SINE PCR performed on 66 F_0_ *An. gambiae* s.l. revealed the presence of 60.61% (40/66) *An. gambiae* s.s. and 39.39% (26/66) *An. coluzzii*. Hence *An. funestus* s.s. was the dominant malaria vector at the beginning of the peak of the major raining season while *An. gambiae* s.l. dominated at the peak of the minor raining season.

### *Plasmodium* infection rates

The head/thoraxes and abdomen of 120 *An. funestus* s.s. were screened separately to detect the presence of *Plasmodium* sporozoites and oocysts, respectively. The proportions of oocyst and sporozoite infections were 10.83% (13/120) and 2.5% (3/120) respectively. For the Oocyst infection, 7.5% (9/120) were identified as *P. falciparum* and 3.33% (4/120) as either *P. ovale, P. vivax*, or *P. malariae* (OVM). On contrary, all the sporozoite infections (3/120) were due to *P. falciparum* (2.5%). Nested PCR confirmed that (6/9) of the *Plasmodium*-infected oocyst were *P. falciparum* and 3/4 of the OVM were due to *P. malariae.* Regarding the *Plasmodium* sporozoite infection, 2 out of the 3 detected were confirmed to be due to *P. falciparum*.

For *An. gambiae* s.l., 41 samples were dissected into head/thoraxes and abdomen. TaqMan revealed an oocyst infection rate of 14.63% (6/41) with 66.67% due to *P. falciparum* and 33.33% OVM. A sporozoite infection rate of 9.76% (4/41) due to *P. falciparum* was obtained. Interestingly, it was also noted that all the *Plasmodium* infections were recorded in *An. gambiae* s.s. while no infection was observed in *An. coluzzii*. Nested PCR recorded no infection from the four sporozoite positive samples detected by TaqMan and revealed similar results with oocyst infection obtained by TaqMan. Also, the nested PCR further identified the 2/41 OVM samples as *P. malariae*.

### Insecticide resistance profile at diagnostic insecticide concentrations

**Fig. 2:**
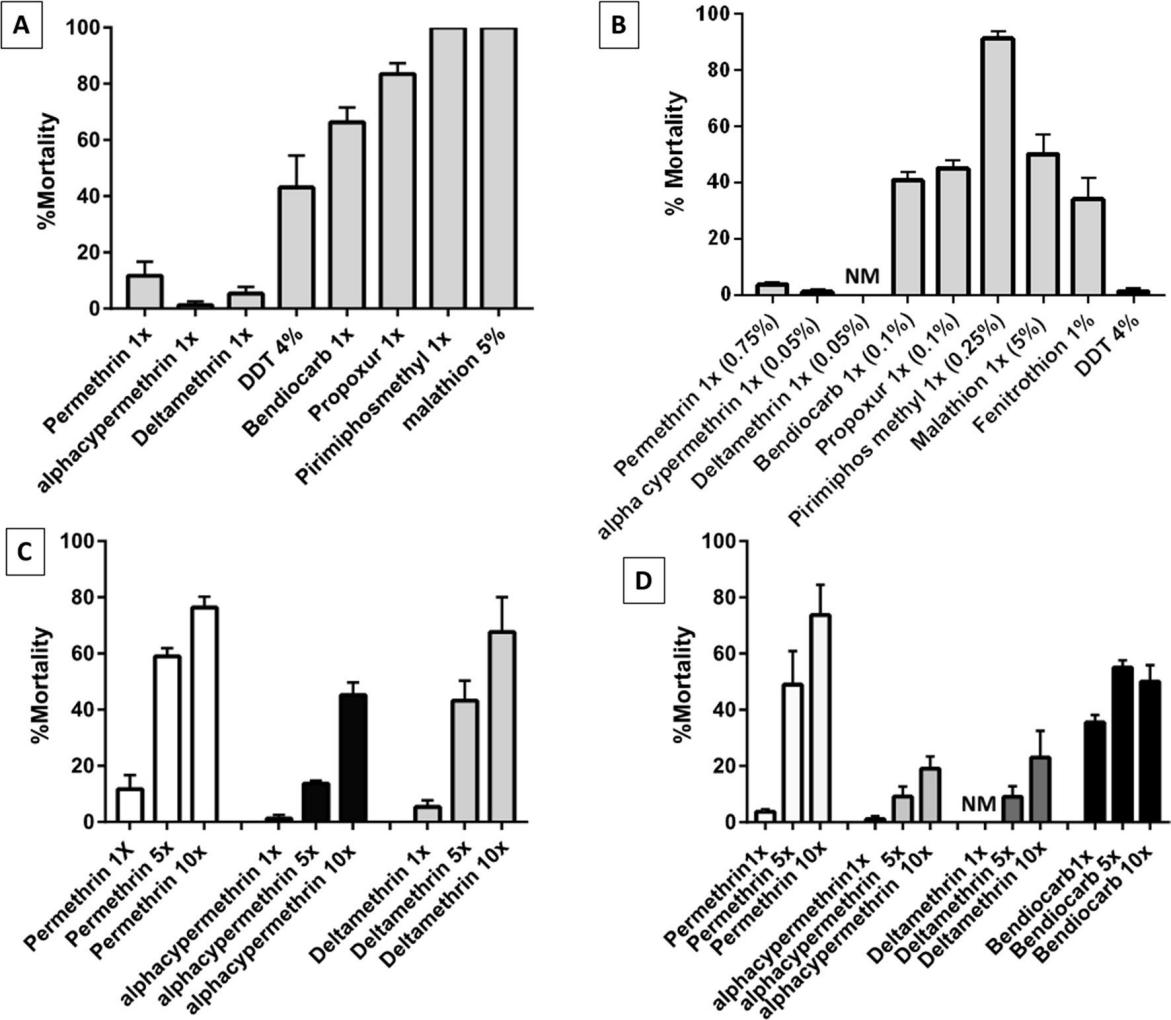
Insecticide susceptibility profile of *Anopheles* species collected in Atatam exposed to 1x, 5 × and 10 × concentrations of insecticides **A** Percentage mortality of *Anopheles funestus* 1 × for various insecticides **B** Percentage mortality of *Anopheles gambiae* s.l. 1 × for various insecticides **C** Resistance intensity of *Anopheles funestus* under 1x, 5 × and 10 × doses. **D** Resistance intensity of *Anopheles gambiae* s.l*.* under 1x, 5 × and 10 × doses

*An. funestus* s.s. from Atatam were resistant to three classes of insecticides used in public health including pyrethroids [both type I (permethrin) and type II (deltamethrin and alphacypermethrin)], organochlorine (DDT), and carbamates (bendiocarb and propoxur) but fully susceptible to organophosphates (pirimiphos methyl and malathion) at standard diagnostic doses (1X). The lowest mortalities were observed for pyrethroids with mortality rates of 1.25 ± 1.25% for alpha-cypermethrin 1x, 11.6 ± 5% for permethrin 1 × and 5.33 ± 2.37% for deltamethrin 1x. DDT had a lower mortality rate of 43.09 ± 11.34% compared to the carbamates, bendiocarb 1x (66.3 ± 5.18%), and propoxur (83.47 ± 3.81%) (Fig. 2A). On the other hand, *An. gambiae* s.l. was found resistant to all four main classes of insecticides used in public health. *An. gambiae* s.l. was highly resistant to pyrethroids and organochloride DDT with mortality ranging from 0–3.7% and 1.25% respectively (Fig. 2B). This *An. gambiae* s.l. population was also resistant to organophosphates including malathion and fenitrothion with mortality rates of 50 ± 7% and 34.06 ± 7.67% respectively (Fig. 2B) while a possible resistance to pirimiphos methyl with a mortality rate of 91.13 ± 2.64% was observed. The mortality rates for the carbamates, bendiocarb and propoxur were 40.73 ± 2.86% and 45.0 ± 3.10% respectively (Fig. 2B).

### Intensity of resistance to pyrethroids and carbamates

The resistance intensity was determined for the pyrethroids (permethrin, alpha-cypermethrin, and deltamethrin) and the carbamate bendiocarb. *An. funestus* s.s. population from Atatam were resistant to both 5 × and 10 × doses of permethrin, deltamethrin, and alpha-cypermethrin hence showing a high-intensity resistance with mortality rates below 90% at 10 × doses of 76.35 ± 3.87%, 67.61 ± 12.45% and 45.17 ± 4.54% respectively (Fig. 2C). At 5 × dose, mortality rates were below 50% for deltamethrin (13.68 ± 1.01%), alpha-cypermethrin (43.19 ± 7.1%) while permethrin had a higher mortality rate (58.95 ± 2.95).

*An. gambiae* s.l. also showed high resistance intensity for the 3 insecticides at higher doses. *An. gambiae* s.l. was more resistant to type II pyrethroids (alpha-cypermethrin and deltamethrin) than type I pyrethroids (permethrin) with higher mortalities scored with an increase in permethrin concentration (Fig. 2D). Permethrin gave mortality rates of 3.70 ± 0.96% at 1x, 48.99 ± 11.85 at 5 × and 73.71 ± 10.75% at 10 × while alpha-cypermethrin and deltamethrin gave 1.09 ± 1.09% and 0% at 1x; 9.12 ± 3.56% and 9.0 ± 3.8% at 5 × and 19.05 ± 4.31% and 23.00 ± 9.5% at 10x. An increase in bendiocarb dose was associated with a slight increase in mortality from 35.56 ± 2.59% at 1 × to 55.01 ± 2.58% at 5 × and a further 50.02 ± 5.84% at 10 × revealing a high resistance intensity (Fig. 2D).

### PBO synergist-insecticide bioassay

**Fig. 3:**
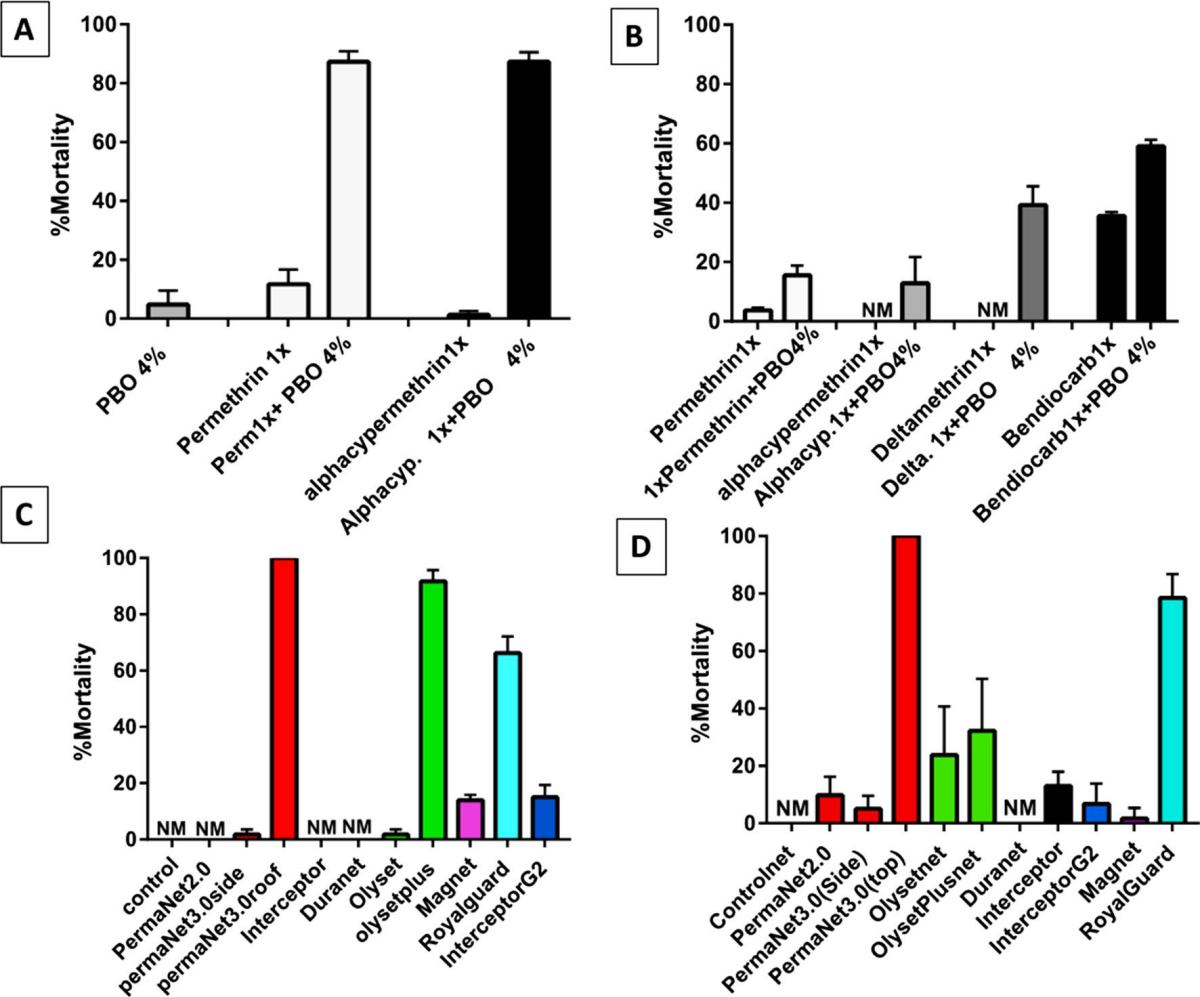
PBO Synergist bioassay and cone assay results. **A** Proportion mortality of *Anopheles funestus* to pyrethroids when pre-exposed to PBO synergist **B** Proportion mortality of *Anopheles gambiae* s.l. to pyrethroids when pre-exposed to PBO synergist **C** Bio-efficacy of different LLINs *An. funestus* s.s. mosquitoes from Atatam using WHO cone bioassays **D** Bio-efficacy of different LLINs against *An. gambiae* s.l. mosquitoes from Atatam using WHO cone bioassays

A significant recovery of susceptibility was observed when *An. funestus* s.s. mosquitoes were pre-exposed to the synergist piperonyl butoxide (PBO) before exposure to permethrin 1 × and alpha-cypermethrin 1x. Overall, the recoveries for permethrin and alpha-cypermethrin were 75.66% and 85.95% respectively indicating partial involvement of cytochrome P450 enzymes (Fig. 3A). Inhibition of cytochrome P450 enzymes in *An. gambiae* s.l. by pre-exposure to PBO before insecticide had a limited impact on the resistance profile. Pre-exposure gave a recovery rate of 11.86% for permethrin, 12.87% for alpha-cypermethrin, 39.20% for deltamethrin, and 23.45% for bendiocarb (Fig. 3B) showing that cytochrome P450s could be playing a minor role in the resistance observed alongside the implication of other enzymes or resistance mechanisms.

### Insecticide-treated bed net bio-efficacy

The effectiveness of some LLINs was determined following the WHO guidelines for cone assays. For *An. funestus* s.s., the assays revealed a loss of efficacy of conventional bed nets (PermaNet 2.0, Interceptor, DuraNet, Olyset, MagNet) with mortalities ranging from 0 to 13.94 ± 1.93% (Fig. 3C). The PBO-based nets were the most effective with mortality rates above 80%. Surprisingly, the novel dual ingredient nets (Royal guard and interceptor G2) had mortality rates below 80% (Fig. 3C).

Similar results were obtained for *An. gambiae* s.l. with reduced efficacy for conventional bed nets like PermaNet 2.0 (9.83 ± 6.41%), Olyset (23.82 ± 16.93%), DuraNet (0%), Interceptor (13.04 ± 4.90%) and MagNet (1.67 ± 8.27%) (Fig. 3D). Only, PermaNet 3.0 net showed high efficacy with 100% mortality while the other PBO-based net Olyset plus gave low mortality of 32.20 ± 18.07%. The novel nets surprisingly showed a reduced efficacy with mortality below 80%. Interceptor G2 had remarkably low mortality of 6.82 ± 7.03% 72 h post bioassays while Royal Guard scored the second-highest mortality (78.47 ± 8.27%) after PermaNet 3.0 (Fig. 3D). All the Nets used in this study were also tested against the *An. gambiae* s.s. Kisumu laboratory susceptible strain which resulted in 100% mortalities.

### Detection and allelic distribution of insecticide resistance markers in field samples

**Fig. 4:**
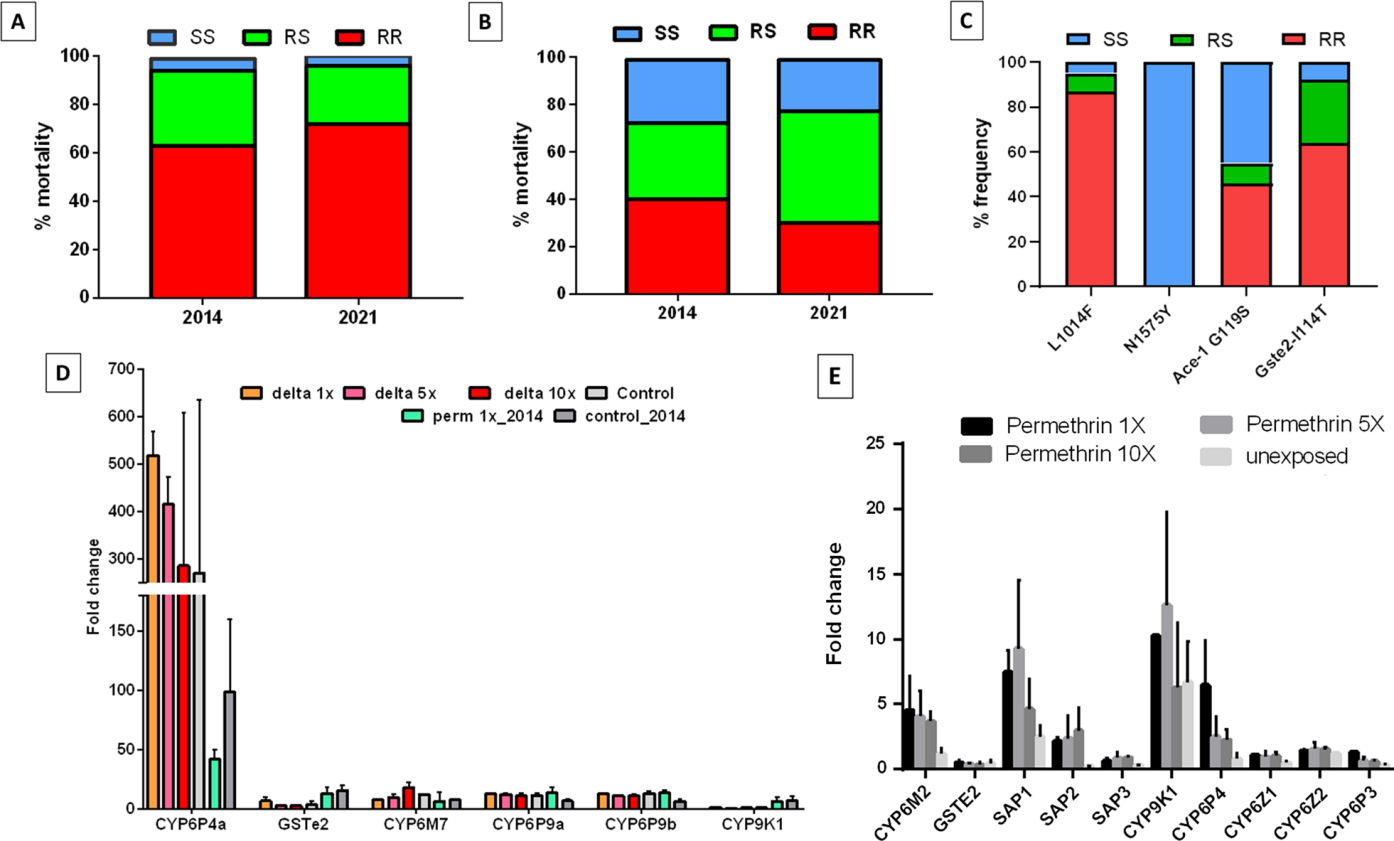
Temporal monitoring of percentage frequency of resistance markers and expression profile of candidate genes **A** A296S mutation associated with resistance to dieldrin and **B** L119F-GSTe2 mutation associated with DDT/pyrethroid resistance **C** Frequencies of resistance markers (kdrW-L1014F, N1575Y, Ace-1 G119S, Gste2-I114T) in An. gambiae **D** Differential expression of candidate resistance genes by qPCR between Deltamethrin 1x, 5 × and 10 × *An. funestus* s.s. resistant mosquitoes, unexposed relative to FANG susceptible lab strain. **E** Differential expression of candidate resistance genes by qPCR between permethrin 1x, 5 × and 10 × *An.gambiae* s.l. resistant mosquitoes, unexposed relative to Kisumu susceptible lab strain

Genotyping of 53 F_0_ *An. funestus* s.s. for both target site and metabolic resistance markers detected resistant genotypes only for A296S-RDL and L119F-*GSTe2* while the *CYP6P9a, CYP6P9b,* and N485I-*Ace* were completely susceptible. The 296S-RDL resistant allele was present at a high frequency of 84% close to what was reported in 2014 (78.95%) [10]. In terms of genotypes, 72% of the individuals genotyped were RR (36/50), 4% were SS (2/50) and 24% RS (12/50) similar to the frequencies reported in 2014 (Fig. 4A).

Detection of the L119F-*GSTe2* mutations associated with DDT/pyrethroid resistance in 53 F_0_ non-exposed mosquitoes revealed a similar trend to A296S-RDL with the *GSTe2* 119F resistance allele present at 53.77%. Genotype distribution detected 30.19% RR (16/53) individuals, 47.17% (25/53) RS and 22.64% SS. Overall the frequencies of the L119F-GSTe2 mutation were similar to those obtained in 2014 both at the allelic and genotypic levels [10] (Fig. 4B).

For *An. gambiae* s.l., the target-site resistance markers (Kdr-west/Kdr-East, N1575Y, and *Ace-1*), as well as the metabolic resistance marker (*GSTe2* I114T), were genotyped in 38 F_0_. The 1014F_resistant allele was found at a high frequency (90.79%) while the 1014S resistant allele was absent (Fig. 4C; Additional file 1: Table S1). The N1575Y mutation was completely absent in the subsamples used. The 119S *Ace-1* resistant marker associated with resistance to carbamates and organophosphates was detected at 50% and genotypic frequencies of 45.71% (16/35) RR, 8.57% (3/35) RS and 45.71% (16/35) SS. At the species level, the 119S frequency was higher in *An. gambiae* s.s. 64% versus 13.63% in *An. coluzzii* (Fig. 4C; Additional file 1: Table S1**).** Genotyping of the *Gste2*-I114T showed that the 114 T resistant allele was present at 77.78% with 63.89% (23/36) RR, 27.78% (10/36) RS, and 8.33% (3/36) SS.

### Role of L119F*-GSTe2* mutations in DDT and escalation of pyrethroid resistance in *An. funestus* s.s.

**Fig. 5:**
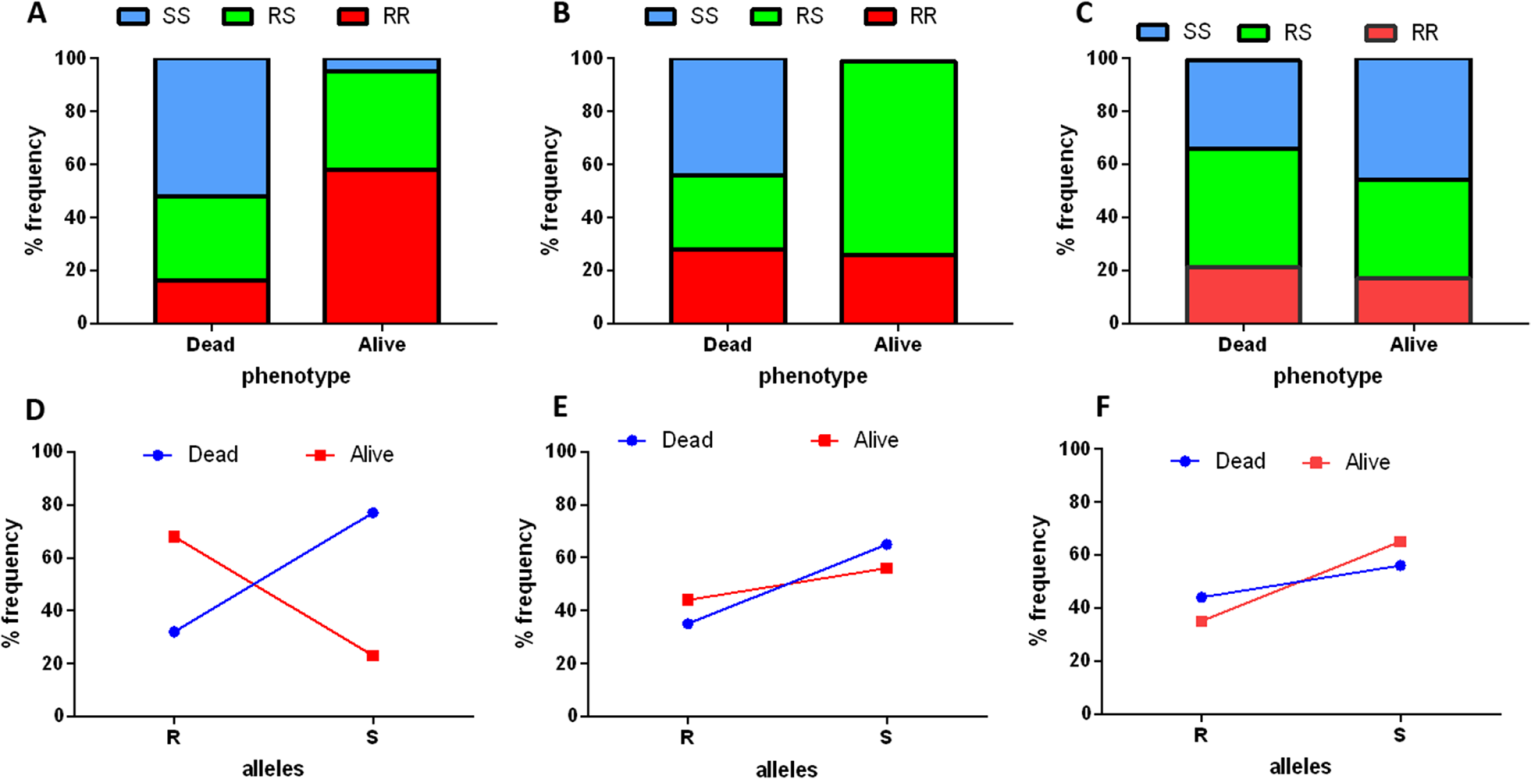
Association of the L119F *GSTe2* mutation with insecticide resistance phenotype. Genotypic frequencies of L119F *GSTe2* resistance mutation among F1 *An. funestus* s.s. mosquitoes exposed to **A** DDT, **B** deltametrhin 5x, and **C** deltamethrin 10x. Allelic frequencies of L119F GSTe2 resistance mutation among F1 *An. funestus* s.s. mosquitoes exposed to **D** DDT, **E** deltamethrin 5X and **F**) deltamethrin 10x

Genotyping of the L119F *GSTe2* in 38 dead and 43 alive F_1_ samples exposed to DDT showed that the 119F allele was present at a higher frequency of 76.74% in survivors compared to the dead mosquitoes (31.57%) while the L119 allele was present at 23.26% in the survivors versus 68.42% in the dead mosquitoes (Additional file 1: Table S2). This revealed an association between the 119F mutation and the ability to survive exposure to DDT (OR: 7.02; 95% CI: 3.75–13.16; P < 0.0001) (Fig. 5A, D). A comparison of genotypes also showed a higher frequency of 58.14% (25/43) for the F/F in alive mosquitoes while the L/L were more in the dead samples at 52.63% (20/38) (Additional file 1: Table S2). There were no significant differences in the L/F genotype among the dead (31.58%) or alive (37.20%) groups. The observed association was stronger in the homozygote state than in the heterozygote state (OR: 9.38; 95% CI: 1.85–47.52; P = 0.0069) indicating an additive effect. F/F and F/L genotypes also showed higher association with the survival ability post-exposure to DDT compared to the L/L with odds ratios of 41.67 (95% CI: 7.57–229.21; P < 0.0001) and 13.33 (95% CI: 2.60–68.39; P = 0.0019) respectively.

The contribution of the L119F *GSTe2* in the escalation of pyrethroid resistance was checked by genotyping this marker in dead and alive mosquitoes exposed to deltamethrin 1x, 5 × and 10 × concentrations. Due to the low mortality rate of 5.33 ± 2.37% at 1 × with only 3 mosquitoes dead, it was not possible to check the correlation at this dose. Using the 5 × samples showed an association with deltamethrin resistance at this increased dose, with a predominance of the 119F allele (63.24%) among the alive compared to the L119 allele (36.76%) (OR; 2.35; 95% CI: 1.33–4.15; P = 0.0032). Genotypic frequencies showed that all the alive were either F/F (26.47%) or L/F (73.52%) while all the L/L were among the dead (19/43). F/F and L/F genotypes were associated with greater chances of surviving 5 × deltamethrin exposure than the L/L with odd ratios of 26.64 (95% CI: 1.58–555.75; P = 0.0234) and 79.56 (95% CI: 4.43–1428.10) respectively (Fig. 5B, E). Detection of the L119F among the 10X deltamethrin alive and dead samples revealed no clear association of this mutation with the ability to survive exposure to a higher dose as the results obtained were not significant (Fisher exact test: 0.247; P < 0.05) (Fig. 5C, F). The 119F allele was present at a higher frequency in the dead (43.93%) than alive (35.42%) samples while surprisingly the L119 allele was present more in the alive (64.58%) than dead (56.06%) samples but this was at non-significant levels.

### Role of G119S *Ace-1* mutations in carbamate and organophosphate resistance in *An. gambiae* s.l.

**Fig. 6:**
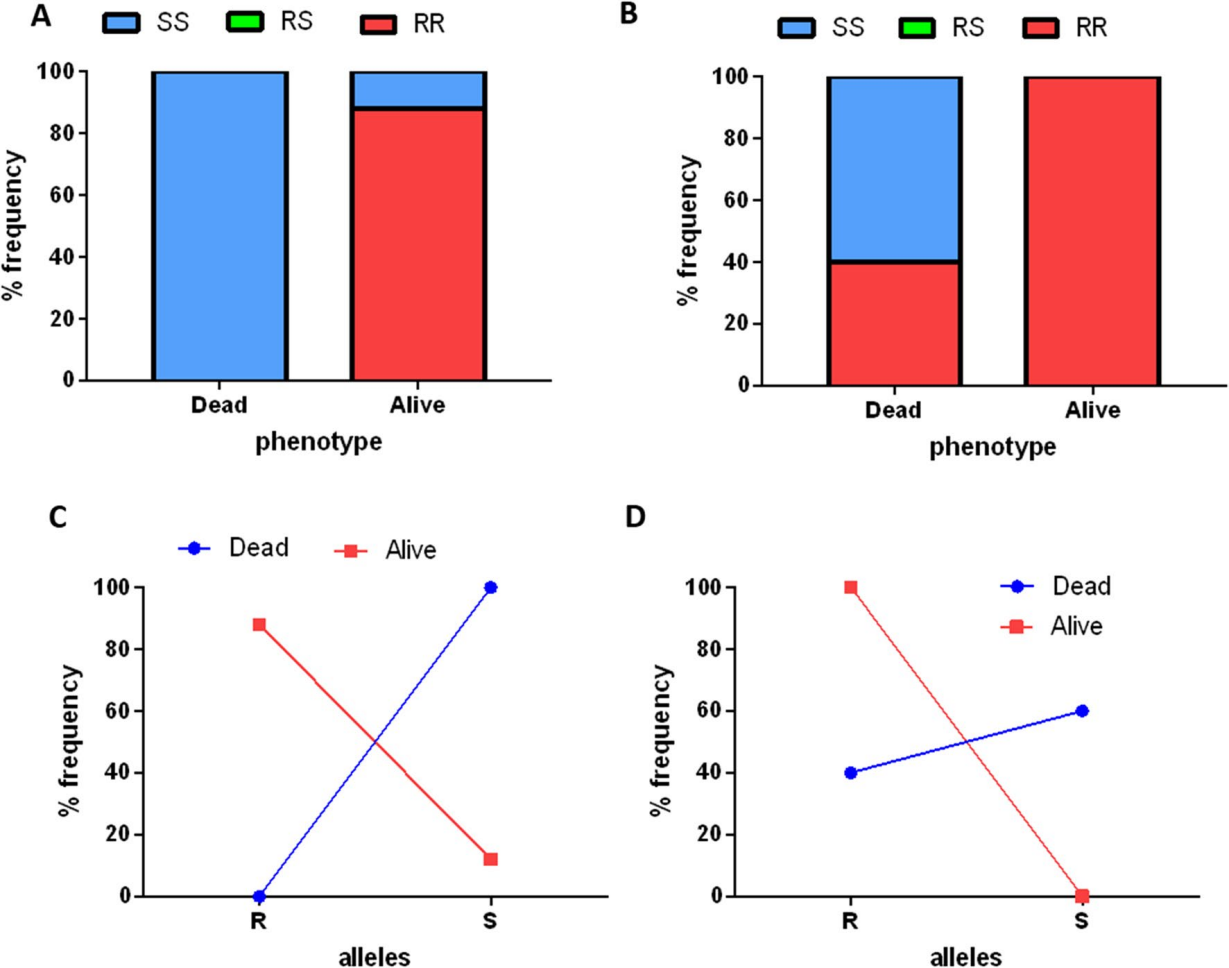
Association of the *Ace-1* G119S mutation with insecticide resistance phenotype. Distribution of *Ace-1* G119S resistance mutation genotypes among F1 *An. gambiae* s.l. mosquitoes exposed to **A** Bendiocarb, and **B** pirimiphos methyl. **C** and **D** show the allelic distribution of G119S mutation among mosquitoes exposed to **C** bendiocarb and **D** pirimiphos methyl

Eighty-seven (51 alive and 36 dead) F_1_ *An. gambiae* s.l. samples were used to check the association between resistance to the carbamate bendiocarb and the *G119S* mutation in the *Ace-1* gene. The 119S resistant allele was found more in the survivors at 88.24% and completely absent in the dead samples (Additional file 1: Table S2). Some samples carrying the G119 susceptible allele (14.28%; 6/42) were able to survive exposure to bendiocarb, but the majority 85.24% (36/42) died after exposure (Fig. 6C). A similar pattern was seen at the genotypic level with only RR and SS genotypes detected at frequencies of 88.24% and 11.76% respectively (Fig. 6A). Hence the resistance to bendiocarb is associated with the presence of the *119S Ace-1* allele (OR; 511; 95% CI: 27.86–9373.55; P < 0.0001).

The association between resistance to pirimiphos methyl and the G119S *Ace-1* resistance mutation was also investigated using 67 (7 alive and 60 dead) F_1_ *An, gambaie* s.l., obtained from bioassays (Fig. 6B, D). TaqMan results identified 31 119S/119S (RR) of which only 7 (22.58%) had survived exposure to pirimiphos methyl while the remaining 24 (77.42%) were dead. All the G119/G119 (SS) were recorded in the dead following the exposure (Additional file 1: Table S2). Hence, the 119S resistant allele was found to be associated with resistance to pirimiphos methyl (OR = 22.35; 95% CI: 1.22–409.47; P < 0.0001).

### Transcription profile of resistance genes in *An. funestus* s.s.

The expression levels of certain candidate genes associated with insecticide resistance were determined. This included the duplicated CYP genes *CYP6P4a* and *CYP6P9a*/*b*, *CYP6M7*, *GSTe2,* and *CYP9K1* all known to drive pyrethroid resistance in *An. funestus* s.s. populations across Africa. These genes were up-regulated in the survivors exposed to 1 × deltamethrin compared to the susceptible FANG except for *CYP9K1* (Fig. 4D , Additional file 1: Table S3). *CYP6P4a* was the most up-regulated with a massive fold change of 518.17 ± 51.27 followed by *CYP6P9a* (12.40 ± 0.71)*, CYP6P9b* (12.61 ± 0.50)*, CYP6M7* (7.48 ± 0.91), and lastly *GSTe2* (6.51 ± 2.7) (Fig. 4D , Additional file 1: Table S3). There was no significant difference observed between the survivors and unexposed (control) indicating that these genes are constitutively expressed except for *CYP6P4a* (p value = 0.027). Analysis of the expression profile of these genes in samples alive after exposure to 1x, 5 × and 10 × doses showed that higher doses do not have much impact on the expression. Apart from *CYP6P4a,* a comparison of the actual expression profile to the previous study done in 2015 revealed no major change in the expression of *CYP6P9a, CYP6P9b, CYP6M7,* and *GSTe2* with previous data showing 13.85 ± 0.37; 8.52 ± 0.18; 5.62 ± 0.65 and 13.59 ± 2.53 respectively***.***

### Transcription profile of resistance genes in *An. gambiae* s.l.

qRT-PCR was used to estimate the expression of 12 candidate genes relative to the Kisumu susceptible laboratory strain. Several genes were found to be upregulated in *An. gambiae* s.l*.* population from Atatam. Genes upregulated with a fold change greater than 2 were in the order *CYP9K1* > *SAP1* > *CYP6M2*. For the cytochrome P450s, *CYP9K1* and *CYP6M2* were 10 and 4- folds more expressed than in the susceptible Kisumu respectively. Out of the 3 chemosensory proteins, 2 (*SAP1* and *SAP2*) were found to be up-regulated at around 5 and 2 folds respectively. The cytochrome P450 monooxygenases *CYP4G16* and *CYP4G17* were downregulated in the survivor mosquitoes. Some genes (*CYP6M2*, *SAP1,* and *SAP2*) were found to be induced as the expression levels were higher in the exposed compared to the non-exposed group (Fig. 4E , Additional file 1: Table S3). Analysis of the level of expression of these genes as the insecticide dose increased from 1 × to 5 × and 10 × revealed no further change in expression level.

## Discussion

Until now the fight against malaria relies mostly on vector control interventions whose effectiveness is threatened by the rise of insecticide resistance. For adequate actions to be taken, it is important to monitor the evolution of insecticide resistance and assess its impact on the effectiveness of current control tools. Knowledge of the susceptibility profile of local malaria vectors against insecticides can guide in designing and implementing suitable resistance management strategies to preserve the efficacy of the current and future vector control tools, especially in malaria-endemic countries. In this study, *An. funestus* s.s. and *An. gambiae* s.l. population collected in Southern Ghana showed multiple and high-intensity resistance to most insecticides used in public health. The data from this study provide valuable information for the NMCPs which will help in decision making and better implementation of insecticide resistance management.

### Malaria transmission has not abated in Atatam with two major vectors present

*An. funestus* s.s. was found to be the main malaria vector in Atatam at the time of collection which is in accordance with previous studies carried out in this area [8–10]. The role of *An. funestus* s.s. in malaria transmission was highlighted with a *Plasmodium* infection rate of 10.8% and 2.5% for the oocyst and sporozoite infection respectively. The oocyst rate was similar to the 12.5% reported in 2014 for whole mosquitoes [10], and the sporozoite rate of 1.81% reported in 2010 [8]. This indicates no reduction in the infection rates notwithstanding the use of bed nets in the area. Despite this, the oocyst infection rate was lower than that reported in other regions across Africa including Cameroon (15%) [35, 36] and Congo (22.2%) [37]. Similarly, the sporozoite *Plasmodium* infection rate was also lower when compared to those previously report in Nigeria (54.55%) [38], Cameroon (5%) [35, 36], Uganda (4.5%) [13] and Malawi (4.8%) [39]. *P. malariae* and *P. falciparum* were both found at the oocyst stage but only *P. falciparum* was present at the sporozoite stage.

The *Plasmodium* infection rate in *An. gambiae* s.l. (14.28%) was higher than that of *An. funestus* s.s. both at the sporozoite and oocyst rate indicating that both vectors are involved in malaria transmission. This is higher than the infection rate of 4.9 previously reported in Atatam. This was still more than what was observed in other localities in southern Ghana: Tarkwa (4.7%), Ahafo (8.3%), and Akyem (5.03%) [8].

### *An. gambiae *s.l. and *An. funestus* s.s. both exhibit multiple and aggravated resistance to insecticides

This study also characterized the insecticide susceptibility pattern of *An. funestus* s.s. and *An. gambiae* s.l. from Ghana. *An. funestus* s.s. showed a high level of resistance to pyrethroids and moderate resistance to DDT and carbamates. A comparison of bioassay results done in 2014 and 2021 showed an increase in resistance level for permethrin indicated by reduced mortality rates from 36.11 ± 3.87% in 2014 to 11.6 ± 5% in 2021. An increase in susceptibility was observed for DDT (17.86 ± 4.51% in 2014 to 43.09 ± 11.34% in 2021) and bendiocarb (21.88 ± 10.71% in 2014 to 66.3 ± 5.18% in 2021). The resistance level has increased when compared to previous reports, especially for the pyrethroids where mortality rates ranged from 1.25 ± 1.25 to 11.67 ± 5% which is quite concerning as the main vector control tool in Atatam is alpha-cypermethrin impregnated LLINs (MagNet) distributed during the last campaign. Intensity bioassays showed that *An. funestus* s.s. can withstand 5 × and 10 × doses of permethrin, alpha-cypermethrin, and deltamethrin with more than 20% of mosquitoes alive at 10X. This resistance intensity profile is higher than that recently reported in other populations from Eastern Africa [40]. Pre-exposure to PBO synergist greatly increased the efficacy of pyrethroids hence revealing the implication of metabolic resistance more precisely the cytochrome P450s detoxification enzymes. The implication of cytochrome P450s implies that LLINs with PBO could be more effective than the conventional bed nets with insecticides only. This was in line with the cone assays results which tested the bio-efficacy of some commercially available nets against this *An. funestus* s.s. population and revealed that PBO-based bed nets were the most effective even surpassing the novel bed nets (Royal guards and interceptor G2). PBO-based bed nets have been shown to reduce malaria incidence [41] and increase personal protection [42] in areas with pyrethroid resistance. The full susceptibility to organophosphates indicates that IRS with pirimiphos methyl (Actellic 300CS) could also be an option in curbing pyrethroid resistance in this area as previously shown in northern Ghana [43] and western Kenya [44]. Even though a reduced performance of conventional bed nets was observed with cone assays, more studies using either experimental hut or looking at the delayed mortality are needed to assess the impact of such multiple resistance on the effectiveness of insecticide-based interventions.

*An. gambiae* s.l. was highly resistant to all public health insecticides tested with possible resistance to pirimiphos methyl. The mortality due to pyrethroids was extremely low similar to what has been reported in other localities in Ghana [15] and other countries such as Ivory Coast [45], Cameroon [46], Nigeria [47] and Democratic Republic of Congo [48]. The intensity of pyrethroid resistance was higher for type II which is different from the frequent pattern where *An. gambiae* s.l. is often more resistant to type I than type II as observed elsewhere [46] but similar to the results reported by Pwalia et al., in 2017. The intensity of resistance to bendiocarb was also high even at 10 × showing that this population is exhibiting both high intensity and multiple resistance. Synergist assays using PBO indicated the involvement of cytochrome P45Os but with a low recovery rate suggesting that other mechanisms might be involved. The recovery rate with PBO was lower than those previously reported by [15] and [49] who evaluated the synergizing effect of PBO in enhancing the efficacy of pyrethroids. The impact of the multiple and high intensity of resistance of *An. gambiae* s.l. from Atatam on the bio-efficacy of different nets was assessed through cone assays and revealed low mortality, particularly for pyrethroid-only bed nets. This low efficacy of pyrethroid only nets has been reported across Africa by several authors [15, 37]. Pyrethroid-PBO net (PermaNet 3.0) was the most effective suggesting that Pyrethroid-PBO nets could better help in controlling malaria in this region. Epidemiological studies and semi-field trials carried out in Africa have shown that these Pyrethroid-PBO nets provide more protective capacity than pyrethroid-only nets in areas with high intensity of resistance [41, 42, 50].

Previous studies have reported the absence of target-site resistance in *An. funestus* s.s. both in Ghana [9] and across Africa [51]. PBO synergist bioassays result established the involvement of cytochrome P450s with *CYP6P4a, CYP6P9a, CYP6P9b* and *CYP6M7* genes being up-regulated. *CYP6P9a/b* were up-regulated but the expression levels were lower than those reported in Mozambique, southern Africa [12]. The previously identified candidate gene *CYP6P4a* was highly overexpressed at about 12 times higher than what was reported in 2015 by Weedall et al. (2019). Further studies are needed to characterize the role of this gene in insecticide resistance to allow the development of a diagnostic tool to track insecticide resistance in this region. Genotyping for the molecular markers previously identified in the promoters of *CYP6P9a* and *CYP6P9b* genes showed that these samples are susceptible indicating that other mechanisms such as trans-regulatory factors, different from those in southern Africa may be involved. The *GSTe2* was found to be up-regulated (FC: 6.51 ± 2.7) and the 119F mutation was also found at a high frequency which correlated strongly with DDT resistance. The significant correlation between the 119F_*GSte2* allele and the ability to survive exposure to 5 × deltamethrin is an indication that *GSTe2* contributes to resistance escalation but the lack of correlation at 10 × could suggest that other molecular factors are driving a higher level of resistance. Overall, the frequency of *GSTe2* in Atatam is higher than reported in Cameroon (23%) [36, 52], Uganda (10%) [13] and Mozambique (7%) [12]. But lower than the 100% reported in Benin [53]. The frequency of A296S mutation associated with resistance to dieldrin remained constant (80%) even with the discontinued use of dieldrin similar to what is observed in *An. gambiae* [54] thereby posing a limitation on the use of other cyclodienes in vector control. The widespread pyrethroid resistance has led to the development of new LLINs which contain the pyrethroid combined with other chemicals such as PBO, chlorfenapyr or pyriproxyfen. Low mortality was obtained with interceptor G2 but further studies using the WHO tunnel test are needed to confirm its effectiveness as cone assays are not a reliable method to measure the bioefficacy of the chlorfenapyr component [55].

In *An. gambiae* s.l., the vgsc-L1014F mutation associated with pyrethroid and DDT resistance was very high at a frequency of 91.4% approaching fixation like in other localities across Africa which could explain the very high pyrethroid resistance observed in Atatam. The *Ace-1 119S* point mutation characterizing resistance to carbamates and organophosphates was found at a moderate frequency of 50% lower than the 76% frequency previously reported in Accra [15]. Genotyping of this mutation in F_1_ survivors and dead mosquitoes exposed to bendiocarb and pirimiphos methyl confirmed the link between this mutation and resistance with the 119S being strongly associated with resistance to Bendiocarb (OR; 511; 95% CI: 27.86–9373.55; P < 0.0001) than with pirimiphos methyl (OR; 22.35; 95% CI: 1.22–409.47; P < 0.0001). Grau-Bove et al. (2021) recently showed that pirimiphos methyl resistance is due to a combination of the *G119S* non-synonymous mutation and copy number variation (CNV) in the acetylcholinesterase *Ace-1* gene. Unfortunately, the involvement of CNV was not investigated here. The high frequency of the 119S mutation in *An. gambiae* s.s. (64%) compared to *An. coluzzii* could be explained by the fact that *Ace-1* substitution evolved in *An. gambiae* s.s. before introgressing in *An. coluzzii* as suggested by [56]. qRT-PCR results also revealed the overexpression of multiple detoxification genes (*CYP9K1, CYP6M2* and *CYP6P4*) which have also been found up-regulated in resistant mosquitoes from the Ivory Coast [57]. In addition, the sensory appendage proteins *SAP1* and *SAP2* previously shown to be overexpressed in West Africa and to also have a high-affinity binding to pyrethroids were found to be upregulated in this population. The lower expression of cytochrome P450s observed in *An. gambiae* s.l. compared to *An. funestus* s.s. further supports the contrasting resistance mechanisms in these two species and supports the lack of recovery after PBO exposure in *An. gambiae*. This reveals the complexity of insecticide resistance phenotype which involves a combination of multiple mechanisms.

In both species, the link between increased overexpression of known detoxification genes and high pyrethroid resistance was investigated with no differences observed between 1x, 5x, and 10 × correlating with previous results in *An. funestus* s.s. from Uganda [13] but different from what has been reported in *An. coluzzii* from Burkina Faso [58]. This lack in difference could be due to the fixation of resistance mechanisms in field populations due to the strong selective pressure exerted by high bed net coverage [59]. In addition, the frequencies of resistance markers were also determined with no association observed at higher doses. This is a narrow approach that does not take into account the contribution of other genes and target site resistance. Taking all these into account, there can be other factors that need to be investigated to understand the drivers of resistance escalation such as the contribution of other detoxification genes, target site, sequestration, cuticular resistance, or microbiome. Hence high-throughput techniques such as RNA-sequencing or whole genome sequencing could facilitate the identification of the molecular drivers contributing to high-intensity resistance.

## Conclusion

The high and multiple insecticide resistance in *An. funestus* s.s. and *An. gambiae* s.l. reported here represents a serious threat to the fight against malaria in Ghana and calls for action to implement insecticide resistance management strategies. The susceptibility to organophosphates and recovery with PBO suggests that vector control tools with this insecticide class could be more appropriate in this area. Ultimately, future vector control interventions should rely on novel insecticide ingredients other than pyrethroids to mitigate the adverse impact of this escalating and multiple resistance in the major malaria vectors.

## Supplementary Information

**Additional file 1: Table S1.** Frequency of target site mutations characterized in *Anopheles gambiae* (s.l.) F_0_ from Atatam. **Table S2.** Frequency of L119F-*GSTe2* resistance markers among dead and alive *An. funestus* exposed to DDT, deltamethrin 1x, 5x and 10x. **Table S3. **Differential expression of metabolic resistance genes among F1 Deltamethrin 1X, 5x, 10x and unexposed mosquitoes from Atatam compared with the FANG susceptible strain. **Table S4.** Differential expression of metabolic and cuticular resistance genes among the *An. gambiae* sl population from Atatam as compared with the Kisumu susceptible strain.

## Data Availability

All data generated or analysed during this study are included in this published article and its Additional files.

## References

[1] World Health Organization. World Malaria Report 2021. World Health Organization 2021. https://apps.who.int/iris/handle/10665/350147. License: CC BY-NC-SA 3.0 IGO.

[2] World Health Organization. World malaria report 2020: 20 years of global progress and challenges. World Health Organization 2020. https://apps.who.int/iris/handle/10665/337660. License: CC BY-NC-SA 3.0 IGO.

[3] World Health Organization. Global technical strategy for malaria 2016–2030. World Health Organization 2019. https://apps.who.int/iris/handle/10665/176712.

[4] World Health Organization. Global Malaria Programme. Global plan for insecticide resistance management in malaria vectors. World Health Organization 2012. https://apps.who.int/iris/handle/10665/44846.

[5] Knox TB, Juma EO, Ochomo EO, Pates Jamet H, Ndungo L, Chege P (2014). An online tool for mapping insecticide resistance in major Anopheles vectors of human malaria parasites and review of resistance status for the Afrotropical region. Parasit Vectors.

[6] Riveron JM, Tchouakui M, Mugenzi LD, Menze B, Chiang M, Wondji CS. Insecticide resistance in malaria vectors: an update at a global scale. In: Manguin S, Dev V, editors. Towards Malaria Elimination - A Leap Forward [Internet]. London: IntechOpen; 2018 [cited 2022 Oct 22]. Available from: https://www.intechopen.com/chapters/62169; 10.5772/intechopen.78375.

[7] Cohuet A, Harris C, Robert V, Fontenille D (2010). Evolutionary forces on Anopheles: what makes a malaria vector?. Trends Parasitol.

[8] Hunt RH, Fuseini G, Knowles S, Stiles-Ocran J, Verster R, Kaiser ML, Choi KS, Koekemoer LL, Coetzee M. Insecticide resistance in malaria vector mosquitoes at four localities in Ghana, West Africa. Parasites Vectors. 2011;4(1):1–7. 10.1186/1756-3305-4-107 PMC3145582 21679391

[9] Okoye PN, Brooke BD, Koekemoer LL, Hunt RH, Coetzee M (2008). Characterisation of DDT, pyrethroid and carbamate resistance in *Anopheles funestus* from Obuasi, Ghana. Trans R Soc Trop Med Hyg.

[10] Riveron JM, Osae M, Egyir-Yawson A, Irving H, Ibrahim SS, Wondji CS (2016). Multiple insecticide resistance in the major malaria vector *Anopheles funestus* in southern Ghana: implications for malaria control. Parasit Vectors.

[11] Mugenzi LMJ, Menze BD, Tchouakui M, Wondji MJ, Irving H, Tchoupo M (2019). Cis-regulatory CYP6P9b P450 variants associated with loss of insecticide-treated bed net efficacy against *Anopheles funestus*. Nat Commun.

[12] Riveron JM, Huijben S, Tchapga W, Tchouakui M, Wondji MJ, Tchoupo M (2019). Escalation of pyrethroid resistance in the malaria vector *Anopheles funestus* induces a loss of efficacy of piperonyl butoxide-based insecticide-treated nets in Mozambique. J Infect Dis.

[13] Tchouakui M, Mugenzi LMJ, Menze BD, Khaukha JNT, Tchapga W, Tchoupo M (2021). Pyrethroid resistance aggravation in ugandan malaria vectors is reducing bednet efficacy. Pathogens.

[14] Hemingway J, Ranson H, Magill A, Kolaczinski J, Fornadel C, Gimnig J (2016). Averting a malaria disaster: will insecticide resistance derail malaria control?. Lancet.

[15] Pwalia R, Joannides J, Iddrisu A, Addae C, Acquah-Baidoo D, Obuobi D (2019). High insecticide resistance intensity of *Anopheles gambiae* (s.l.) and low efficacy of pyrethroid LLINs in Accra, Ghana. Parasites Vectors.

[16] Asare-Nuamah P, Botchway E (2019). Comparing smallholder farmers’ climate change perception with climate data: the case of Adansi North District of Ghana. Heliyon.

[17] Gillies MT, Coetzee M. A supplement to the Anophelinae of Africa South of the Sahara. Publ S Afr Inst Med Res. 1987;55:1–43.

[18] Morgan JC, Irving H, Okedi LM, Steven A, Wondji CS (2010). Pyrethroid resistance in an *Anopheles funestus* population from uganda. PLoS ONE.

[19] Livak KJ (1984). Organization and mapping of a sequence on the Drosophila melanogaster X and Y chromosomes that is transcribed during spermatogenesis. Genetics.

[20] Koekemoer LL, Kamau L, Hunt RH, Coetzee M (2002). A cocktail polymerase chain reaction assay to identify members of the *Anopheles funestus* (Diptera: Culicidae) group. Am J Trop Med Hyg.

[21] Santolamazza F, Mancini E, Simard F, Qi Y, Tu Z, della Torre A. (2008). Insertion polymorphisms of SINE200 retrotransposons within speciation islands of *Anopheles gambiae* molecular forms. Malar J.

[22] Bass C, Nikou D, Blagborough AM, Vontas J, Sinden RE, Williamson MS (2008). PCR-based detection of Plasmodium in Anopheles mosquitoes: a comparison of a new high-throughput assay with existing methods. Malar J.

[23] Snounou G, Viriyakosol S, Zhu XP, Jarra W, Pinheiro L, do Rosario VE (1993). High sensitivity of detection of human malaria parasites by the use of nested polymerase chain reaction. Mol Biochem Parasitol.

[24] World Health Organization. Test procedures for insecticide resistance monitoring in malaria vector mosquitoes, 2nd edn. World Health Organization 2016. https://apps.who.int/iris/handle/10665/250677.

[25] World Health Organization & WHO Pesticide Evaluation Scheme. Guidelines for laboratory and field-testing of long-lasting insecticidal nets. World Health Organization 2013. https://apps.who.int/iris/handle/10665/80270.

[26] Tchouakui M, Chiang MC, Ndo C, Kuicheu CK, Amvongo-Adjia N, Wondji MJ (2019). A marker of glutathione S-transferase-mediated resistance to insecticides is associated with higher Plasmodium infection in the African malaria vector *Anopheles funestus*. Sci Rep.

[27] Weedall GD, Mugenzi LMJ, Menze BD, Tchouakui M, Ibrahim SS, Amvongo-Adjia N (2019). A cytochrome P450 allele confers pyrethroid resistance on a major African malaria vector, reducing insecticide-treated bednet efficacy. Sci Transl Med.

[28] Ibrahim SS, Ndula M, Riveron JM, Irving H, Wondji CS (2016). The P450 CYP6Z1 confers carbamate/pyrethroid cross-resistance in a major African malaria vector beside a novel carbamate-insensitive N485I acetylcholinesterase-1 mutation. Mol Ecol.

[29] Martinez-Torres D, Chandre F, Williamson MS, Darriet F, Bergé JB, Devonshire AL (1998). Molecular characterization of pyrethroid knockdown resistance (kdr) in the major malaria vector *Anopheles gambiae* s.s.. Insect Mol Biol.

[30] Ranson H, Jensen B, Vulule JM, Wang X, Hemingway J, Collins FH (2000). Identification of a point mutation in the voltage-gated sodium channel gene of Kenyan *Anopheles gambiae* associated with resistance to DDT and pyrethroids. Insect Mol Biol.

[31] Jones CM, Liyanapathirana M, Agossa FR, Weetman D, Ranson H, Donnelly MJ (2012). Footprints of positive selection associated with a mutation (N1575Y) in the voltage-gated sodium channel of *Anopheles gambiae*. Proc Natl Acad Sci U S A.

[32] Ingham VA, Anthousi A, Douris V, Harding NJ, Lycett G, Morris M (2019). A sensory appendage protein protects malaria vectors from pyrethroids. Nature.

[33] Riveron JM, Irving H, Ndula M, Barnes KG, Ibrahim SS, Paine MJI (2013). Directionally selected cytochrome P450 alleles are driving the spread of pyrethroid resistance in the major malaria vector *Anopheles funestus*. Proc Natl Acad Sci U S A.

[34] Schmittgen TD, Livak KJ (2008). Analyzing real-time PCR data by the comparative CT method. Nat Protoc.

[35] Nkemngo FN, Mugenzi LMJ, Tchouakui M, Nguiffo-Nguete D, Wondji MJ, Mbakam B (2022). Xeno-monitoring of molecular drivers of artemisinin and partner drug resistance in P. falciparum populations in malaria vectors across Cameroon. Gene.

[36] Menze BD, Wondji MJ, Tchapga W, Tchoupo M, Riveron JM, Wondji CS (2018). Bionomics and insecticides resistance profiling of malaria vectors at a selected site for experimental hut trials in central Cameroon. Malar J.

[37] Riveron JM, Watsenga F, Irving H, Irish SR, Wondji CS (2018). High plasmodium infection rate and reduced bed net efficacy in multiple insecticide-resistant malaria vectors in Kinshasa, Democratic Republic of Congo. J Infect Dis.

[38] Ibrahim SS, Mukhtar MM, Irving H, Riveron JM, Fadel AN, Tchapga W (2020). Exploring the mechanisms of multiple insecticide resistance in a highly plasmodium-infected malaria vector *Anopheles funestus* sensu stricto from Sahel of northern Nigeria. Genes (Basel).

[39] Riveron JM, Chiumia M, Menze BD, Barnes KG, Irving H, Ibrahim SS (2015). Rise of multiple insecticide resistance in *Anopheles funestus* in Malawi: a major concern for malaria vector control. Malar J.

[40] Tchouakui M, Mugenzi LMJ, Wondji MJ, Tchoupo M, Njiokou F, Wondji CS (2021). Combined over-expression of two cytochrome P450 genes exacerbates the fitness cost of pyrethroid resistance in the major African malaria vector *Anopheles funestus*. Pestic Biochem Physiol.

[41] Staedke SG, Gonahasa S, Dorsey G, Kamya MR, Maiteki-Sebuguzi C, Lynd A (2020). Effect of long-lasting insecticidal nets with and without piperonyl butoxide on malaria indicators in Uganda (LLINEUP): a pragmatic, cluster-randomised trial embedded in a national LLIN distribution campaign. Lancet.

[42] Martin JL, Mosha FW, Lukole E, Rowland M, Todd J, Charlwood JD (2021). Personal protection with PBO-pyrethroid synergist-treated nets after 2 years of household use against pyrethroid-resistant Anopheles in Tanzania. Parasit Vectors.

[43] Coleman S, Yihdego Y, Sherrard-Smith E, Thomas CS, Dengela D, Oxborough RM (2021). Partial indoor residual spraying with pirimiphos-methyl as an effective and cost-saving measure for the control of *Anopheles gambiae* s.l. in northern Ghana. Sci Rep.

[44] Abong’o B, Gimnig JE, Torr SJ, Longman B, Omoke D, Muchoki M (2020). Impact of indoor residual spraying with pirimiphos-methyl (Actellic 300CS) on entomological indicators of transmission and malaria case burden in Migori County, western Kenya. Sci Rep.

[45] Kouassi BL, Edi C, Tia E, Konan LY, Akré MA, Koffi AA (2020). Susceptibility of *Anopheles gambiae* from Côte d’Ivoire to insecticides used on insecticide-treated nets: evaluating the additional entomological impact of piperonyl butoxide and chlorfenapyr. Malar J.

[46] Piameu M, Nwane P, Toussile W, Mavridis K, Wipf NC, Kouadio PF (2021). Pyrethroid and etofenprox resistance in *Anopheles gambiae* and anopheles coluzzii from vegetable farms in yaoundé, cameroon: dynamics, intensity and molecular basis. Molecules.

[47] Oyeniyi AT, Adeogun AO, Idowu ET, Oboh B, Olakiigbe A, Adesalu O (2020). First report of N1575Y mutation in pyrethroid resistant *Anopheles gambiae* s.l. in Nigeria. Sci Afr.

[48] Wat’senga F, Agossa F, Manzambi EZ, Illombe G, Mapangulu T, Muyembe T (2020). Intensity of pyrethroid resistance in *Anopheles gambiae* before and after a mass distribution of insecticide-treated nets in Kinshasa and in 11 provinces of the Democratic Republic of Congo. Malar J.

[49] Dadzie SK, Chabi J, Asafu-Adjaye A, Owusu-Akrofi O, Baffoe-Wilmot A, Malm K (2017). Evaluation of piperonyl butoxide in enhancing the efficacy of pyrethroid insecticides against resistant *Anopheles gambiae* s.l. in Ghana. Malar J.

[50] Protopopoff N, Mosha JF, Lukole E, Charlwood JD, Wright A, Mwalimu CD (2018). Effectiveness of a long-lasting piperonyl butoxide-treated insecticidal net and indoor residual spray interventions, separately and together, against malaria transmitted by pyrethroid-resistant mosquitoes: a cluster, randomised controlled, two-by-two fact. Lancet.

[51] Irving H, Wondji CS (2017). Investigating knockdown resistance (kdr) mechanism against pyrethroids/DDT in the malaria vector *Anopheles funestus* across Africa. BMC Genet.

[52] Nkemngo FN, Mugenzi LMJ, Terence E, Niang A, Wondji MJ, Tchoupo M (2020). Elevated Plasmodium sporozoite infection and multiple insecticide resistance in the principal malaria vectors *Anopheles funestus* and *Anopheles gambiae* in a forested locality close to the Yaoundé airport, Cameroon [version 1; peer review: 1 approved, 1 app. Wellcome Open Res.

[53] Riveron JM, Yunta C, Ibrahim SS, Djouaka R, Irving H, Menze BD (2014). A single mutation in the GSTe2 gene allows tracking of metabolically based insecticide resistance in a major malaria vector. Genome Biol.

[54] Grau-Bové X, Tomlinson S, O’Reilly AO, Harding NJ, Miles A, Kwiatkowski D (2020). Evolution of the insecticide target Rdl in african anopheles is driven by interspecific and interkaryotypic introgression. Mol Biol Evol.

[55] Lissenden N, Armistead JS, Gleave K, Irish SR, Martin JL, Messenger LA, Moore SJ, Ngufor C, Protopopoff N, Oxborough R, Spiers A. Developing consensus standard operating procedures (SOPs) to evaluate new types of insecticide-treated nets. Insects, 2021;13(1):7. 10.3390/insects13010007 PMC8778287 35055850

[56] Grau-Bové X, Lucas E, Pipini D, Rippon E, Van’t Hof AE, Constant E (2021). Resistance to pirimiphos-methyl in West African Anopheles is spreading via duplication and introgression of the Ace1 locus. PLoS Genet..

[57] Oumbouke WA, Pignatelli P, Barreaux AMG, Tia IZ, Koffi AA, Ahoua Alou LP (2020). Fine scale spatial investigation of multiple insecticide resistance and underlying target-site and metabolic mechanisms in *Anopheles gambiae* in central Côte d’Ivoire. Sci Rep.

[58] Toé KH, N’Falé S, Dabiré RK, Ranson H, Jones CM (2015). The recent escalation in strength of pyrethroid resistance in Anopheles coluzzi in West Africa is linked to increased expression of multiple gene families. BMC Genomics.

[59] Barnes KG, Weedall GD, Ndula M, Irving H, Mzihalowa T, Hemingway J (2017). Genomic footprints of selective sweeps from metabolic resistance to pyrethroids in African Malaria vectors are driven by scale up of insecticide-based vector control. PLoS Genet.

