# Supplementary material for: Escalating pyrethroid resistance in two major malaria vectors Anopheles funestus and Anopheles gambiae (s.l.) in Atatam, Southern Ghana
Source: BMC Infect Dis. 2022 Oct 25;22:799. doi: 10.1186/s12879-022-07795-4 (PMC9597992; doi:10.1186/s12879-022-07795-4)
Supplement: Supplementary file 1 — Additional file 1: Table S1. Frequency of target site mutations characterized in Anopheles gambiae (s.l.) F0 from Atatam. Table S2. Frequency of L119F-GSTe2 resistance markers among dead and alive An. funestus exposed to DDT, deltamethrin 1x, 5x and 10x. Table S3. Differential expression of metabolic resistance genes among F1 Deltamethrin 1X, 5x, 10x and unexposed mosquitoes from Atatam compared with the FANG susceptible strain. Table S4. Differential expression of metabolic and cuticular resistance genes among the An. gambiae sl population from Atatam as compared with the Kisumu susceptible strain. [file 12879_2022_7795_MOESM1_ESM.docx]

**Table S1: Frequency of target site mutations characterized in *Anopheles gambiae* (s.l.) F_0_ from Atatam**

| **Insecticide** | **# Mosquitoes genotyped** | **% Homozygote mutation (RR)** | **% Heterozygote mutation (RS)** | **% Homozygote wild type (SS)** | **R** | **S** |
| --- | --- | --- | --- | --- | --- | --- |
| ***L1014F*** | 38 | 86.84(33) | 7.89(3) | 5.26(2) | 90.79 | 9.21 |
| ***N1575Y*** | 38 | 0 | 0 | 100 | 0 | 100 |
| ***Ace-1 G119S*** | 35 | 45.71(16) | 8.57(3) | 45.71(16) | 50 | 50 |
| ***Gste2-*I114T** | 36 | 63.89(23) | 27.78(10) | 8.33(3) | 77.78 | 22.22 |

**Table S2: Frequency of L119F-*GSTe2* resistance markers among dead and alive *An. funestus* exposed to DDT, deltamethrin 1x, 5x and 10x**

| **Insecticide** | **Survival status** | **# Mosquitoes genotyped** | **% Homozygote mutation (RR)** | **% Heterozygote mutation (RS)** | **% Homozygote wild type (SS)** | **L119F allele frequency** | | **Fischer exact test** | ***P value*** |
| --- | --- | --- | --- | --- | --- | --- | --- | --- | --- |
|  |  |  |  |  |  | **R** | **S** |  |  |
| **DDT** | Alive | 43 | 58.14 (25) | 37.20 (16) | 4.65(2) | 76.74 | 23.26 | 40.83 | < 0.001 |
|  | Dead | 38 | 15.79 (6) | 31.58 (12) | 52.63 (20) | 31.58 | 68.42 |  |  |
| **Deltamethrin 1X** | Alive | 48 | 33.33 (16) | 52.08 (25) | 14.58 (7) | 50 | 50 | 1.8493 | 0.174 |
|  | Dead | 3 | 33.33 (1) | 33.33 (1) | 33.33 (1) | 59.38 | 40.63 |  |  |
| **Deltamethrin 5X** | Alive | 34 | 26.47 (9) | 73.53 (25) | 44.19 (0) | 63.24 | 36.76 | 20.5922 | < 0.001 |
|  | Dead | 43 | 27.91 (12) | 27.91 (12) | 44.19 (19) | 41.86 | 58.14 |  |  |
| **Deltamethrin 10X** | Alive | 24 | 16.67 (4) | 37.5 (9) | 45.83 (11) | 35.4167 | 64.58 | 1.69 | 0.193 |
|  | Dead | 66 | 21.21 (14) | 45.45 (30) | 33.33 (22) | 43.94 | 56.06 |  |  |

**Table S3.** Differential expression of metabolic resistance genes among F1 Deltamethrin 1X, 5x, 10x and unexposed mosquitoes from Atatam compared with the FANG susceptible strain

|  | **Gene** | **Fold change Vs FANG** | **P-Value** |
| --- | --- | --- | --- |
| 1x Deltamethrin alive | *CYP6P4a* | 255.31±61.67 | 0.015 |
|  | *GSTe2* | 4.16±0.43 | 0.004 |
|  | *CYP6M7* | 8.98±1.08 | 0.002 |
|  | *CYP6P9a* | 10.23±2.84 | 0.023 |
|  | *CYP6P9b* | 11.10±1.37 | 0.001 |
|  | *CYP9K1* | 1.21±0.23 | 0.278 |
| 5x Deltamethrin | *CYP6P4a* | 263.18±16.84 | < 0.001 |
|  | *GSTe2* | 2.82±0.33 | 0.003 |
|  | *CYP6M7* | 9.92±2.84 | 0.027 |
|  | *CYP6P9a* | 11.75±1.90 | 0.004 |
|  | *CYP6P9b* | 12.12±1.99 | 0.004 |
|  | *CYP9K1* | 0.64±0.14 | 0.078 |
| 10x deltamethrin | *CYP6P4a* | 181.53±53.35 | < 0.001 |
|  | *GSTe2* | 4.33±1.80 | 0.001 |
|  | *CYP6M7* | 17.71±3.05 | 0.005 |
|  | *CYP6P9a* | 13.86±3.54 | 0.017 |
|  | *CYP6P9b* | 14.52±1.99 | 0.022 |
|  | *CYP9K1* | 1.33±0.12 | 0.070 |
| Control unexposed | *CYP6P4a* | 277.2±57.47 | 0.010 |
|  | *GSTe2* | 4.19±1.5 | 0.062 |
|  | *CYP6M7* | 8.58±4.58 | 0.106 |
|  | *CYP6P9a* | 8.96±1.81 | 0.012 |
|  | *CYP6P9b* | 11.05±3.26 | 0.027 |
|  | *CYP9K1* | 0.87±0.37 | 0.392 |

**Table S4.** Differential expression of metabolic and cuticular resistance genes among the *An. gambiae* sl population from Atatam as compared with the Kisumu susceptible strain.

|  | **Gene** | **Fold change Vs FANG** | **P-value** |
| --- | --- | --- | --- |
| Permetrin 1x | *CYP6M2* | 4.55± 1.33 | 0.082 |
|  | *GSTE2* | 0.51±0.09 | 0.083 |
|  | *SAP1* | 7.47±0.85 | 0.039 |
|  | *SAP2* | 2.17 ± 0.15 | 0.002 |
|  | *SAP3* | 0.67±0.08 | 0.500 |
|  | *CYP9K1* | 10.2±0.08 | 0.0002 |
|  | *CYP6P4* | 6.47±1.72 | 0.028 |
|  | *CYP6Z1* | 1.12±0.03 | 0.300 |
|  | *CYP6Z2* | 1.43±0.04 | 0.055 |
|  | *CYP6P3* | 1.34±0.033 | 0.144 |
|  | *CYP4G16* | 0.04±0.004 | 0.011 |
|  | *CYP4G17* | 0.1±0.05 | 0.010 |
| Permethrin 5X | *CYP6M2* | 4.06±1 | 0.028 |
|  | *GSTE2* | 0.42±0.06 | 0.138 |
|  | *SAP1* | 9.26±2.62 | 0.063 |
|  | *SAP2* | 2.39±0.08 | 0.120 |
|  | *SAP3* | 0.89±0.22 | 0.312 |
|  | *CYP9K1* | 12.59±3.59 | 0.020 |
|  | *CYP6P4* | 2.53±0.77 | 0.087 |
|  | *CYP6Z1* | 0.98±0.20 | 0.500 |
|  | *CYP6Z2* | 1.59±0.24 | 0.078 |
|  | *CYP6P3* | 0.68±0.13 | 0.130 |
|  | *CYP4G16* | 0.7±0.02 | 0.012 |
|  | *CYP4G17* | 0.16±0.03 | 0.015 |
| Permethrin 10X | *CYP6M2* | 3.69±0.37 | 0.001 |
|  | *GSTE2* | 0.39±0.09 | 0.145 |
|  | *SAP1* | 4.66±1.12 | 0.054 |
|  | *SAP2* | 2.96±0.89 | 0.064 |
|  | *SAP3* | 0.9±0.07 | 0.351 |
|  | *CYP9K1* | 6.28±2.48 | 0.007 |
|  | *CYP6P4* | 2.28±0.38 | 0.031 |
|  | *CYP6Z1* | 1.03±0.16 | 0.418 |
|  | *CYP6Z2* | 1.56±0.08 | 0.020 |
|  | *CYP6P3* | 0.61±0.06 | 0.084 |
|  | *CYP4G16* | 0.04±0.01 | 0.011 |
|  | *CYP4G17* | 0.11±0.02 | 0.012 |
| *Control unexposed* | *CYP6M2* | 1.22±0.21 | 0.458 |
|  | *GSTE2* | 0.48±0.11 | 0.172 |
|  | *SAP1* | 2.52±0.44 | 0.068 |
|  | *SAP2* | 0.23±0.01 | 0.001 |
|  | *SAP3* | 0.32±0.04 | 0.348 |
|  | *CYP9K1* | 6.7±1.57 | 0.014 |
|  | *CYP6P4* | 0.81±0.22 | 0.304 |
|  | *CYP6Z1* | 0.52±0.08 | 0.115 |
|  | *CYP6Z2* | 1.24±0.00 | 0.193 |
|  | *CYP6P3* | 0.34±0.06 | 0.048 |
|  | *CYP4G16* | 0.03±0.01 | 0.032 |
|  | *CYP4G17* | 0.09±0.005 | 0.033 |
